# The Impact of *HMGB1* Polymorphisms on Prostate Cancer Progression and Clinicopathological Characteristics

**DOI:** 10.3390/ijerph17197247

**Published:** 2020-10-03

**Authors:** Ying-Erh Chou, Po-Jen Yang, Chia-Yen Lin, Yen-Yu Chen, Whei-Ling Chiang, Pei-Xuan Lin, Zih-Yun Huang, Matthew Huang, Yung-Chuan Ho, Shun-Fa Yang

**Affiliations:** 1 School of Medicine, Chung Shan Medical University, Taichung 402, Taiwan; intointo814@gmail.com (Y.-E.C.); cshy1030@csh.org.tw (P.-J.Y.); 2 Institute of Medicine, Chung Shan Medical University, Taichung 402, Taiwan; lcyhank.tw@gmail.com; 3 Department of Medical Research, Chung Shan Medical University Hospital, Taichung 402, Taiwan; 4 Department of Family and Community Medicine, Chung Shan Medical University Hospital, Taichung 402, Taiwan; 5 Division of Urology, Department of Surgery, Taichung Veterans General Hospital, Taichung 407, Taiwan; 6 School of Medical Applied Chemistry, Chung Shan Medical University, Taichung 402, Taiwan; ct511c3038@gmail.com; 7 School of Medical Laboratory and Biotechnology, Chung Shan Medical University, Taichung 402, Taiwan; wlchiang@csmu.edu.tw (W.-L.C.); xuan87yte@gmail.com (P.-X.L.); forget0928240084@gmail.com (Z.-Y.H.); 8 White Oaks Secondary School, Oakville, ON L6H 1Z5, Canada; Matthewh0304@gmail.com

**Keywords:** prostate cancer, *HMGB1*, polymorphism

## Abstract

Prostate cancer is one of the major cancers of the genitourinary tract. High-mobility group box 1 (HMGB1) was suggested as a promising therapeutic target for prostate cancer. In this study, we aim to elucidate the associations of *HMGB1* single nucleotide polymorphisms (SNPs) with prostate cancer susceptibility and clinicopathological characteristics. The *HMGB1* SNPs rs1412125, rs2249825, rs1045411, and rs1360485 in 579 prostate cancer patients and 579 cancer-free controls were analyzed with real-time polymerase chain reactions (real-time PCR). All of the data were evaluated with SAS statistical software. Our results showed that the *HMGB1* rs1045411 T allele genotype was significantly associated with advanced pathologic T stage (odds ratio (OR) = 1.433, 95% confidence interval (CI) = 1.021–2.012; *p* = 0.037) and pathologic N1 stage (OR = 2.091, 95% CI = 1.160–3.767; *p* = 0.012), and the rs1360485 polymorphic CT + TT genotype was associated with pathologic Gleason grade group (4 + 5) (OR = 1.583, 95% CI = 1.017–2.462; *p* = 0.041), pathologic T stage (3 + 4) (OR = 1.482, 95% CI = 1.061–2.070; *p* = 0.021), and pathologic N1 stage (OR = 2.131, 95% CI = 1.178–3.852; *p* = 0.011) compared with their wild-type carriers. In conclusion, our results revealed that the *HMGB1* SNPs were associated with the clinical status of prostate cancer. The *HMGB1* SNPs may have the potential to predict prostate cancer disease progression.

## 1. Introduction

Prostate cancer is the second most common cause of male malignancy worldwide [1,2]. In Taiwan, it is the fifth most common cancer, with the seventh-highest cancer-related mortality rate [3]. Aging and high-fat diets have been suggested as epidemiological risk factors that contribute to the rise of prostate cancer incidence in Taiwan [3,4]. Although androgen receptors (ARs) and the androgen signaling axis were the traditional focus for prostate cancer research and clinical therapy development [5,6,7,8], genome-wide assays have identified multiple highly penetrant genes (HPGs) and high-risk single nucleotide polymorphisms (SNPs) associated with prostate cancer risk in the prostate cancer-diagnosed genome [9,10,11]. 

High-mobility group box 1 (*HMGB1*) is a member of the HMG1-type polypeptides and acts as a DNA chaperone, sustaining nucleosome dynamics and chromosomal stability [1,12,13]. In prostate cancer, it was found that targeting *HMGB1* with short hairpin RNA (shRNA) in prostate cancer cells leads to an inhibition of prostate cancer cell survival [14]. Overexpression of *HMGB1* was observed to be significantly associated with poor biochemical recurrence (BCR)-free survival in patients after radical prostatectomy [15]. Moreover, *HMGB1* was suggested as a novel target for potential therapeutics since highly expressed *HMGB1* was found to be associated with the epithelial-to-mesenchymal transition (EMT) and the overexpression of MMP-1, MMP-3, and MMP-10 via the RAGE/NF-κB signaling pathways, facilitating prostate cancer metastasis [1,16,17,18,19,20]. Furthermore, polymorphisms of *HMGB1* have been associated with many cancers, including oral squamous cell carcinoma (OSCC) [21,22], lung cancer [23,24,25,26], breast cancer [27,28,29], gastric cancer [30], hepatocellular carcinoma (HCC) [31,32], and colorectal cancer (CRC) [33], and it was suggested that the SNPs of *HMGB1* may provide a potential biomarker for predicting cancer risk, tumor development, or chemotherapy responses [21,25,27,31]. However, the impact of *HMGB1* polymorphisms on prostate cancer susceptibility and clinicopathologic characteristics has remained uninvestigated. In this study, we focus on four SNPs of *HMGB1*, rs1412125, rs2249825, rs1045411, rs1360485, and try to elucidate their associations with prostate cancer susceptibility and clinicopathologic characteristics.

## 2. Materials and Methods

### 2.1. Study Subjects

In this study, we enrolled 579 patients with prostate cancer as the study group. These patients received robot-assisted radical prostatectomy at Taichung Veteran General Hospital from 2012 to 2017. At the same time, 579 individuals who entered the hospital for a physical examination were selected from the physical examination center to serve as the control group. The control groups were generally healthy without a cancer history and without diseases related to the vital organs according to routine laboratory tests. Participants’ personal characteristics and information, including age, gender, and medical histories, were obtained using interviewer-administered questionnaires. The study was certified by the Institutional Review Broad (IRB) of Taichung Veteran General Hospital, and informed consent was collected from each individual involved in this study (IRB No. CE19062A). Medical information including age at diagnosis, initial prostate specific antigen (PSA) level at diagnosis, pathologic Gleason grade group, clinical and pathological TNM staging, seminal vesicle invasion, perineural invasion, lymphovascular invasion, and D’Amico classification were acquired from each patient’s medical records [34,35,36,37]. In this study, the Gleason grade group and D’Amico classification were grouped according to the International Society of Urological Pathology (ISUP) consensus in 2016 [38].

### 2.2. Sample Preparation and DNA Extraction

The blood specimens were collected from patients with prostate cancer and normal controls involved in our study. The peripheral whole blood samples were preserved in EDTA-containing tubes, followed with centrifugation at a setting of 3000 rpm for 10 min. To collect the DNA, we performed a DNA extraction assay by using QIAamp DNA blood kits. All the protocols of DNA extraction was performed according to the instructions in the manufacturer’s manual [39,40]. The final extracted DNA was applied as a DNA template in the polymerase chain reaction (PCR) assay.

### 2.3. Selection of HMGB1 SNPs

In this study, four SNPs of *HMGB1*, rs1412125 (in the promoter region), rs2249825 (intron 1), rs1045411 (3’UTR), and rs1360485 (3’UTR), were selected from the Single Nucleotide Polymorphism Database (dbSNP) [41]. The SNP rs1412125 of *HMGB1* was selected because the rs1412125 polymorphism was suggested to be associated with pneumonia susceptibility, severity, and inflammatory response [42]. For *HMGB1* rs1360485, patients with one C allele in the rs1360485 domain were suggested to be at greater risk of developing T2 tumors and lymph node metastasis in breast cancer [27]. The SNP rs1045411 of *HMGB1* was selected because it was observed that rs1045411 was correlated with a significant effect on *HMGB1* expression, and was significantly associated with clinical outcomes, especially in Chinese gastric cancer patients with aggressive status after surgery [30].

### 2.4. HMGB1 SNPs Genotyping

The *HMGB1* rs1412125, rs2249825, rs1045411 and rs1360485 SNPs were assayed by using quantitative real-time PCR (qRT-PCR) with an ABI StepOne Real-Time PCR system (Applied Biosystems, Foster City, CA, USA). All the PCR primers and Taqman probes were designed by the same company (assay ID C_8690889_10 for rs1412125; C_15815760_10 for rs2249825; C_3025198_30 for rs1045411; and C_8690872_10 for rs1360485).

### 2.5. Statistical Analyses

To compare the age between controls and the patients with prostate cancer, a Student’s *t*-test was performed. The adjusted odds ratios (AORs) with 95% confidence intervals (CIs) were estimated using a multivariable logistic model after adjusting for age to determine the distribution frequency of *HMGB1* genotypes between control group and prostate cancer patients. For estimating the odds ratio (OR) of the association between the *HMGB1* SNPs and prostate cancer risk, logistic regression models were adopted for data analysis and evaluation. All of the data in our study were evaluated with SAS statistical software. A value of *p* < 0.05 was considered to represent statistical significance, with * standing for *p* < 0.05, ** indicating *p* < 0.01, and *** meaning *p* < 0.001.

## 3. Results

The distribution of demographic characteristics in 579 control individuals and 579 patients with prostate cancer is given in Table 1. In the current study, we observed that the distribution of age at diagnosis (>60 years) was 25.7% (149/579) of the controls and 82.6% (478/579) of the patients with prostate cancer. Two hundred and seventy patients (46.6%) had high PSA levels (PSA > 10 ng/mL), and the median level was 10.54 ng/mL (range 0.634–685). A significant distributional difference was found for age at diagnosis (>60 years) between the controls and patients with prostate cancer (*p* < 0.001).

The distribution frequency of *HMGB1* genotypes in 579 control individuals and 579 patients with prostate cancer is given in Table 2. To diminish the possible interference of age, the adjusted odds ratios (AORs) with 95% CIs were estimated by multiple logistic regression models after controlling for age in each comparison. As shown in Table 2, no significant difference was observed for patients with prostate cancer among the rs1412125, rs2249825, rs1045411, and rs1360485 polymorphisms of the *HMGB1* gene and those with the wild-type (WT) gene in the pre- and post-adjustment for age (Table 2).

To clarify the role of *HMGB1* genetic polymorphisms in prostate cancer status, the clinical status and *HMGB1* genotype frequencies in 579 prostate cancer patients were assessed (Table 3, Table 4 and Table 5). For *HMGB1* rs1412125, the *HMGB1* rs1412125 polymorphic TC + CC genotypic variant was significantly associated with a higher D’Amico classification (OR = 1.405, 95% CI = 1.009–1.956; *p* = 0.044) compared with the TT genotype in 579 patients with prostate cancer (Table 3). The *HMGB1* rs1045411 polymorphic CT + TT genotype was significantly associated with advanced pathologic stage (OR = 1.433, 95% CI = 1.021–2.012; *p* = 0.037) and pathologic N1 stage (OR = 2.091, 95% CI = 1.160–3.767; *p* = 0.012) compared to the wild-type carriers (Table 4). Moreover, the rs1360485 polymorphic TC + CC genotype was associated with the pathologic Gleason grade 4 + 5 group (OR = 1.583, 95% CI = 1.017–2.462; *p* = 0.041), pathologic T3 + T4 stage (OR = 1.482, 95% CI = 1.061–2.070; *p* = 0.021), and pathologic N1 stage (OR = 2.131, 95% CI = 1.178–3.852; *p* = 0.011) compared with the wild-type carriers (Table 5). However, no significant association was found between the *HMGB1* rs2249825 SNPs and clinical status in 579 prostate cancer patients (data not shown).

## 4. Discussion

In this study, we demonstrated the associations between *HMGB1* polymorphisms and prostate cancer. Many risk factors, including age, the concentration of prostate-specific antigen (PSA), family history, insulin-like growth factors, ethnicity, obesity, alcohol consumption, sexually transmitted disease, vasectomy, and smoking, were suggested to be associated with prostate cancer risk [43]. As to the correlations of age and PSA levels with prostate cancer risk, previous studies have suggested that, from the age of 60, the concentration of PSA predicts the lifetime risk of metastasis and death from prostate cancer [43,44,45]. In our study, 478 (82.6%) of 579 prostate cancer patients were diagnosed at age > 60 (*p* < 0.001, Table 1), while more of the patients involved in our study had a PSA level at diagnosis of ≤ 10 ng/mL (53.4%, Table 1). Therefore, it seems that age > 60 is a more dominant risk factor for those individuals involved in our study.

We further examined the associations between *HMGB1* polymorphisms and prostate cancer susceptibility. However, no significant associations were found among *HMGB1* rs1412125, rs2249825, rs1045411, rs1360485, and prostate cancer (Table 2), suggesting that the direct impact of *HMGB1* SNPs on prostate cancer susceptibility might be limited. Intriguingly, after we analyzed the associations of clinical status and these *HMGB1* SNPs, we found that the *HMGB1* rs1412125 polymorphic variants were associated with a higher D’Amico classification (*p* = 0.044, Table 3). Moreover, the *HMGB1* rs1045411 genetic variant CT + TT genotype was found to have statistically significant associations in pathologic T stage (stage 3 + 4, *p* = 0.037) and pathologic N stage (stage N1, *p* = 0.012) compared with the wild-type carriers (Table 4). Similar results were also observed in *HMGB1* rs1360485, while *HMGB1* rs1360485 TC + CC polymorphic variants were found to have statistically significant associations with pathologic Gleason grade (group 4 + 5, *p* = 0.041), pathologic T stage (stage 3 + 4, *p* = 0.021), and pathologic N stage (stage N1, *p* = 0.011) compared to their wild-type carriers (Table 5). Taken together, these results suggested that the SNPs of *HMGB1* might play a more crucial role in tumor progression than carcinogenesis and cancer susceptibility in prostate cancer.

The role of *HMGB1* SNPs in cancer risk, disease progression, and tumor development remains controversial. Some studies have associated *HMGB1* SNPs with increased cancer risk, disease susceptibility, severity, and progression, or poorer response to treatment [22,23,27,31,33,42,46,47,48,49]. However, other studies have indicated that the *HMGB1* SNPs are associated with a lower risk of cancer and a less invasive disease [26,50], or may even not be associated with the risk of cancer [51]. Of note, no information on *HMGB1* polymorphisms and prostate cancer risk was mentioned or discussed in the meta-analysis conducted by Li et al. [51], so the correlations of *HMGB1* SNPs with prostate cancer risk and disease progression remain uncertain. We found no statistically significant association between the *HMGB1* polymorphic variants and prostate cancer patients, implying that the direct impact of *HMGB1* SNPs on prostate cancer susceptibility might be limited (Table 2). However, the results of our study have demonstrated that the *HMGB1* polymorphisms rs1412125, rs1045411, and rs1360485 were associated with prostate cancer disease progression and severity (Table 3, Table 4 and Table 5), suggesting more consistency of *HMGB1* SNPs in the oncogenicity of various cancers. In contrast, we found that the *HMGB1* rs2249825 polymorphic variants showed no significant association with prostate cancer susceptibility (Table 2) or the clinical status of prostate cancer, suggesting a difference from colorectal cancer [33] and cervical cancer [47]. Besides, our previous study found that urothelial cell carcinoma (UCC) patients who carry at least one T allele of the *HMGB1* rs1045411 polymorphism had a lower risk and less invasive disease [50]. However, in our current study, the *HMGB1* rs1045411 polymorphic variants were associated with advanced pathologic T stage (*p* = 0.037) and pathologic N1 stage (*p* = 0.012) in prostate cancer patients (Table 4). One possible reason for this phenomenon is that rs1045411 is located in the 3′ untranslated region of *HMGB1*, which is the most sensitive region to microRNA epigenetic regulation [50]. Thus, the increased susceptibility of malignant progression might result from *HMGB1* mRNA instability [30,50,52]. Taken together, these results exhibit the variety of *HMGB1* SNPs in different cancers, consistent with the oncogenic and tumor-suppressing dual roles of *HMGB1* during tumor development and therapy [13]. Different ethnicities, epigenetic factors, or genetic susceptibility might be responsible for this phenomenon [53,54]. A limitation of our study is that we lack data on *HMGB1* mRNA or protein expression in prostate cancer patients, so more detailed analyses and evaluations could not be performed.

## 5. Conclusions

In conclusion, our study demonstrated that the *HMGB1* SNPs were associated with prostate cancer progression and development. Patients who carried the *HMGB1* rs1412125, rs1045411, and rs1360485 polymorphic variants were associated with a poorer prognosis of prostate cancer, including a higher D’Amico classification, higher pathologic Gleason grade group, higher pathologic T stage, and higher pathologic N stage. The *HMGB1* polymorphisms may serve as a predictor of prostate cancer development and tumor progression.

## Figures and Tables

**Table 1:** The distributions of demographical characteristics in 579 control individuals and 579 patients with prostate cancer.

Variable	Controls (*n* = 579) (%)	Patients (*n* = 579) (%)	*p*-Value
**Age at diagnosis (years)**			
≤60	430 (74.3%)	101 (17.4%)	*p* < 0.001
>60	149 (25.7%)	478 (82.6%)	
Mean ± S.D.	52.31 ± 10.08	67.08 ± 7.42	*p* < 0.001
**PSA at diagnosis (ng/mL)**			
≤10		309 (53.4%)	
>10		270 (46.6%)	
**Pathologic Gleason grade group**			
1 + 2 + 3		484 (83.6%)	
4 + 5		95 (16.4%)	
**Clinical T stage**			
1 + 2		501 (86.5%)	
3 + 4		78 (13.5%)	
**Pathologic T stage**			
2		306 (52.8%)	
3 + 4		273 (47.2%)	
**Pathologic N stage**			
N0		530 (91.5%)	
N1		49 (8.5%)	
**Seminal vesicle invasion**			
No		452 (78.1%)	
Yes		127 (21.9%)	
**Perineural invasion**			
No		155 (26.8%)	
Yes		424 (73.2%)	
**Lymphovascular invasion**			
No		482 (83.2%)	
Yes		97 (16.8%)	
**D’Amico classification**			
Low risk		60 (10.4%)	
Intermediate risk		220 (38.0%)	
High risk		299 (51.6%)	

**Table 2:** Distribution frequency of high-mobility group box 1 (HMGB1) genotypes in 579 control individuals and 579 patients with prostate cancer.

Variables	Controls (*n* = 579) (%)	Patients (*n* = 579) (%)	OR (95% CI) †	AOR (95% CI) ‡
**rs1412125**				
TT	318 (54.9%)	333 (57.5%)	1.00	1.00
TC	222 (38.3%)	209 (36.1%)	0.899 (0.705–1.147)	0.757 (0.562–1.021)
CC	39 (6.8%)	37 (6.4%)	0.906 (0.563–1.457)	0.626 (0.354–1.108)
TC + CC	261 (45.1%)	246 (42.5%)	0.900 (0.714–1.135)	0.848 (0.649–1.107)
**rs2249825**				
CC	431 (74.4%)	438 (75.6%)	1.00	1.00
CG	139 (24.0%)	126 (21.8%)	0.892 (0.677–1.175)	0.820 (0.587–1.146)
GG	9 (1.6%)	15 (2.6%)	1.640 (0.710–3.788)	1.785 (0.647–4.925)
CG + GG	148 (25.6%)	141 (24.4%)	0.937 (0.718–1.223)	0.874 (0.632–1.208)
**rs1045411**				
CC	353 (61.0%)	367 (63.4%)	1.00	1.00
CT	200 (34.5%)	186 (32.1%)	0.895 (0.698–1.146)	0.887 (0.656–1.199)
TT	26 (4.5%)	26 (4.5%)	0.962 (0.548–1.689)	0.847 (0.429–1.672)
CT + TT	226 (39.0%)	212 (36.6%)	0.902 (0.711–1.144)	0.882 (0.661–1.178)
**rs1360485**				
TT	335 (57.9%)	347 (59.9%)	1.00	1.00
TC	211 (36.4%)	199 (34.4%)	0.911 (0.713–1.163)	0.900 (0.668–1.213)
CC	33 (5.7%)	33 (5.7%)	0.965 (0.582–1.600)	0.897 (0.486–1.657)
TC + CC	244 (42.1%)	232 (40.1%)	0.918 (0.726–1.160)	0.899 (0.676–1.196)

† The odds ratios (ORs) with their 95% confidence intervals were estimated by logistic regression models. ‡ The adjusted odds ratios (AORs) with their 95% confidence intervals were estimated by multiple logistic regression models after controlling for age.

**Table 3:** Odds ratios (ORs) and 95% confidence intervals (CIs) of clinical status and *HMGB1* rs1412125 genotypic frequencies in 579 patients with prostate cancer.

Variable	Genotypic Frequencies			
rs1412125	TT (*N* = 333)	TC + CC (*N* = 246)	OR (95% CI)	*p*-Value
**PSA at diagnosis (ng/mL)**				
≤10	151 (45.3%)	119 (48.4%)	1.00	*p* = 0.470
>10	182 (54.7%)	127 (51.6%)	0.885 (0.636–1.232)	
**Pathologic Gleason grade group**				
1 + 2 + 3	282 (84.7%)	202 (82.1%)	1.00	*p* = 0.409
4 + 5	51 (15.3%)	44 (17.9%)	1.204 (0.774–1.874)	
**Clinical T stage**				
1 + 2	284 (85.3%)	217 (88.2%)	1.00	*p* = 0.308
3 + 4	49 (14.7%)	29 (11.8%)	0.775 (0.474–1.267)	
**Pathologic T stage**				
2	183 (55.0%)	123 (50.0%)	1.00	*p* = 0.238
3 + 4	150 (45.0%)	123 (50.0%)	1.220 (0.877–1.697)	
**Pathologic N stage**				
N0	307 (92.2%)	223 (90.7%)	1.00	*p* = 0.510
N1	26 (7.8%)	23 (9.3%)	1.218 (0.677–2.190)	
**Seminal vesicle invasion**				
No	260 (78.1%)	192 (78.0%)	1.00	*p* = 0.993
Yes	73 (21.9%)	54 (22.0%)	1.002 (0.673–1.492)	
**Perineural invasion**				
No	88 (26.4%)	67 (27.2%)	1.00	*p* = 0.828
Yes	245 (73.6%)	179 (72.8%)	0.960 (0.662–1.392)	
**Lymphovascular invasion**				
No	282 (84.7%)	200 (81.3%)	1.00	*p* = 0.281
Yes	51 (15.3%)	46 (18.7%)	1.272 (0.821–1.970)	
**D’Amico classification**				
Low/Intermediate risk	173 (52.0%)	107 (43.5%)	1.00	*p* = 0.044 *
High risk	160 (48.0%)	139 (56.5%)	1.405 (1.009–1.956)	

The ORs and their 95% CIs were estimated by logistic regression models. * *p*-value < 0.05 = statistically significant.

**Table 4:** Odds ratios (ORs) and 95% confidence intervals (CIs) of clinical status and *HMGB1* rs1045411 genotypic frequencies in 579 patients with prostate cancer.

Variable	Genotypic Frequencies			
rs1045411	CC (*N* = 367)	CT + TT (*N* = 2 12)	OR (95% CI)	*p*-Value
**PSA at diagnosis (ng/mL)**				
≤10	167 (45.5%)	103 (48.6%)	1.00	*p* = 0.474
>10	200 (54.5%)	109 (51.4%)	0.884 (0.630–1.240)	
**Pathologic Gleason grade group**				
1 + 2 + 3	314 (85.6%)	170 (80.2%)	1.00	*p* = 0.093
4 + 5	53 (14.4%)	42 (19.8%)	1.464 (0.937–2.286)	
**Clinical T stage**				
1 + 2	321 (87.5%)	180 (84.9%)	1.00	*p* = 0.385
3 + 4	46 (12.5%)	32 (15.1%)	1.241 (0.763–2.018)	
**Pathologic T stage**				
2	206 (56.1%)	100 (47.2%)	1.00	*p* = 0.037 *
3 + 4	161 (43.9%)	112 (52.8%)	1.433 (1.021–2.012)	
**Pathologic N stage**				
N0	344 (93.7%)	186 (87.7%)	1.00	*p* = 0.012 *
N1	23 (6.3%)	26 (12.3%)	2.091 (1.160–3.767)	
**Seminal vesicle invasion**				
No	294 (80.1%)	158 (74.5%)	1.00	*p* = 0.118
Yes	73 (19.9%)	54 (25.5%)	1.376 (0.921–2.056)	
**Perineural invasion**				
No	103 (28.1%)	52 (24.5%)	1.00	*p* = 0.354
Yes	264 (71.9%)	160 (75.5%)	1.200 (0.815–1.768)	
**Lymphovascular invasion**				
No	312 (85.0%)	170 (80.2%)	1.00	*p* = 0.134
Yes	55 (15.0%)	42 (19.8%)	1.401 (0.900–2.183)	
**D’Amico classification**				
Low/Intermediate risk	187 (51.0%)	93 (43.9%)	1.00	*p* = 0.100
High risk	180 (49.0%)	119 (56.1%)	1.329 (0.946–1.867)	

The ORs and their 95% CIs were estimated by logistic regression models. * *p*-value < 0.05 = statistically significant.

**Table 5:** Odds ratios (ORs) and 95% confidence intervals (CIs) of clinical status and *HMGB1* rs1360485 genotypic frequencies in 579 patients with prostate cancer.

Variable	Genotypic Frequencies			
rs1360485	TT (*N* = 347)	TC + CC (*N* = 232)	OR (95% CI)	*p*-Value
**PSA at diagnosis (ng/mL)**				
≤10	161 (46.4%)	109 (47.0%)	1.00	*p* = 0.890
>10	186 (53.6%)	123 (53.0%)	0.977 (0.700–1.363)	
**Pathologic Gleason grade group**				
1 + 2 + 3	299 (86.2%)	185 (79.7%)	1.00	*p* = 0.041 *
4 + 5	48 (13.8%)	47 (20.3%)	1.583 (1.017–2.462)	
**Clinical T stage**				
1 + 2	302 (87.0%)	199 (85.8%)	1.00	*p* = 0.664
3 + 4	45 (13.0%)	33 (14.2%)	1.113 (0.686–1.805)	
**Pathologic T stage**				
2	197 (56.8%)	109 (47.0%)	1.00	*p* = 0.021 *
3 + 4	150 (43.2%)	123 (53.0%)	1.482 (1.061–2.070)	
**Pathologic N stage**				
N0	326 (93.9%)	204 (87.9%)	1.00	*p* = 0.011 *
N1	21 (6.1%)	28 (12.1%)	2.131 (1.178–3.852)	
**Seminal vesicle invasion**				
No	279 (80.4%)	173 (74.6%)	1.00	*p* = 0.096
Yes	68 (19.6%)	59 (25.4%)	1.399 (0.941–2.081)	
**Perineural invasion**				
No	97 (28.0%)	58 (25.0%)	1.00	*p* = 0.431
Yes	250 (72.0%)	174 (75.0%)	1.164 (0.797–1.700)	
**Lymphovascular invasion**				
No	296 (85.3%)	186 (80.2%)	1.00	*p* = 0.105
Yes	51 (14.7%)	46 (19.8%)	1.435 (0.926–2.226)	
**D’Amico classification**				
Low/Intermediate risk	178 (51.3%)	102 (44.0%)	1.00	*p* = 0.084
High risk	169 (48.7%)	130 (56.0%)	1.342 (0.961–1.875)	

The ORs and their 95% CIs were estimated by logistic regression models. * *p*-value < 0.05 = statistically significant.
